# SUMOylation Regulates the Transcriptional Repression Activity of FOG-2 and Its Association with GATA-4

**DOI:** 10.1371/journal.pone.0050637

**Published:** 2012-11-30

**Authors:** José Perdomo, Xing-Mai Jiang, Daniel R. Carter, Levon M. Khachigian, Beng H. Chong

**Affiliations:** 1 Department of Medicine, St George Clinical School, University of New South Wales, Sydney, New South Wales, Australia; 2 Centre for Vascular Research, University of New South Wales, Sydney, New South Wales, Australia; 3 Children’s Cancer Institute Australia, Lowy Cancer Research Centre, University of New South Wales, Sydney, New South Wales, Australia; 4 Haematology Department, St George and Sutherland Hospitals, University of New South Wales, Sydney, New South Wales, Australia; North Carolina State University, United States of America

## Abstract

Friend of GATA 2 (FOG-2), a co-factor of several GATA transcription factors (GATA-4, -5 and 6), is a critical regulator of coronary vessel formation and heart morphogenesis. Here we demonstrate that FOG-2 is SUMOylated and that this modification modulates its transcriptional activity. FOG-2 SUMOylation occurs at four lysine residues (K312, 471, 915, 955). Three of these residues are part of the characteristic SUMO consensus site (ψKXE), while K955 is found in the less frequent TKXE motif. Absence of SUMOylation did not affect FOG-2′s nuclear localization. However, mutation of the FOG-2 SUMOylation sites, or de-SUMOylation, with SENP-1 or SENP-8 resulted in stronger transcriptional repression activity in both heterologous cells and cardiomyocytes. Conversely, increased FOG-2 SUMOylation by overexpression of SUMO-1 or expression of a SUMO-1-FOG-2 fusion protein rendered FOG-2 incapable of repressing GATA-4-mediated activation of the B-type natriuretic peptide (BNP) promoter. Moreover, we demonstrate both increased interaction between a FOG-2 SUMO mutant and GATA-4 and enhanced SUMOylation of wild-type FOG-2 by co-expression of GATA-4. These data suggest a new dynamics in which GATA-4 may alter the activity of FOG-2 by influencing its SUMOylation status.

## Introduction

The cardiac development program involves a number of transcriptional regulators. One essential organizer of cardiogenesis is the transcription factor GATA-4, which recognises the consensus WGATAR motif, found in many cardiac promoters. Many studies have implicated GATA-4 in heart development processes. For instance, it is involved in the differentiation of progenitors into beating cardiac cells *in vitro* [1], and in heart tube formation and yolk sac development *in vivo* [2]. Moreover GATA-4 is required for the expression of cardiac structural genes such as troponin, atrial natriuretic factor (ANF), B-type natriuretic peptide (BNP) and α and β myosin heavy chain (MHC) [3]. Despite the physical association of GATA-4 with several co-factors [3], it is its interaction with the multi-zinc finger protein Friend of GATA 2 (FOG-2) that appears to be crucial for its cardiac function [4].

FOG-2 is a multi-zinc finger protein that, like the related haematopoietic factor FOG-1, operates as a co-factor of GATA proteins. FOG-2 is expressed with GATA-4, -5 and -6 in both the developing and adult heart and the generation of a FOG-2 deficient mouse demonstrated that it is essential for heart morphogenesis and proper cardiovascular development [5]. The phenotype of FOG-2^−/−^ mice was recapitulated to a large extent by a GATA-4 knock-in animal that expresses a GATA-4 molecule that fails to interact with FOG-2 [4], suggesting that FOG-2 is indispensable for GATA-4 activity. Typically, FOG-2 acts as a repressor of GATA-4-mediated activation but could also be a transcriptional activator depending on the cellular and promoter context [6]. GATA-4 is functionally involved in cardiac hypertrophy [7] and is required for the hypertrophic response *in vivo* [8]. FOG-2 is capable of counteracting this effect and protecting cultured cardiac cells against hypertrophy [9]. The mechanism by which FOG-2 modulates GATA-4 activity is yet to be fully elucidated. It is known, however, that FOG-2 interacts functionally with the co-repressor CtBP in *Xenopus* embryos [10] and in cellular assays [11], but this interaction appears to be dispensable for the cardiac-specific ANF promoter examined by Svensson et al [12]. In addition, there is evidence that the N-terminal domain of FOG-2 constitutes an independent NuRD-interacting repression domain [12], [13]. Importantly, this region is conserved in FOG-1, where it serves as a docking domain for the NuRD complex, and is necessary for FOG-1/GATA-1-mediated transcriptional repression [14]. Additionally, FOG-2 may repress transcription by competing directly with GATA-4 for binding to the co-activator p300 [9].

In addition to protein-protein interactions, the function of many transcription factors is altered by post-translational modifications such as phosphorylation, ubiquitination and SUMOylation. Modification by the Small Ubiquitin-related Modifier (SUMO) leads to diverse effects depending on the substrate modified [15]. SUMOylation is a dynamic modification in which a SUMO moiety is covalently added, in an enzymatic process, to target lysine residues within the consensus site ψKXE (where ψ is large and hydrophobic and X is any amino acid). The SUMOylation pathway consists of an E1 activating enzyme (the SAE1/SAE2 heterodimer) and an E2 conjugating enzyme (Ubc9) which transfers the SUMO molecule to the target residue [16]. While E1 and E2 enzymes are sufficient for the SUMOylation of substrates *in vitro*, specific SUMO E3 ligases and de-SUMOylating enzymes have also been described [17].

SUMOylation of transcriptional regulators often contributes to their ability to repress gene expression [15], [18]. For instance, mutation of the SUMOylation site of the repressor BKLF resulted in elimination of its repression activity [19]. In addition, the lack of SUMO modification of several activators, including Sp3 [20] and p300 [21] renders them more potent activators, suggesting that SUMOylation confers a repressive attribute to these molecules. In contrast, lack of SUMO modification reduced the ability of FOG-1 to transactivate the c-mpl promoter [22] and rendered Ikaros a more potent repressor of transcription [23].

Here we report that FOG-2 SUMOylation is necessary for the biological activity of FOG-2. We show that endogenous FOG-2 is SUMOylated and localized the SUMO acceptor sites between zinc fingers 2 and 3, 4 and 5, and 7 and 8, at lysines 324, 471, 915 and 955. Mutation of these residues completely abolishes FOG-2 SUMOylation. Our data indicate that SUMOylation functions to inhibit the capacity of FOG-2 to repress GATA-4-mediated activation. As such, mutant FOG-2 incapable of SUMOylation demonstrates enhanced repression activity, and de-SUMOylation of FOG-2 by SENP1 or SNEP-8 also increases FOG-2-mediated repression. We propose that the enhanced repression activity in the absence of SUMOylation is due to a higher affinity physical interaction between FOG-2 and GATA-4.

## Materials and Methods

### Plasmid Constructs

The expression vector for mouse FOG-2 (accession AF107306), pCS2+FOG-2, [24] was kindly provided by Alan Cantor (Children's Hospital, Boston, MA). Site-directed mutagenesis was performed using the QuikChange Mutagenesis kit (Stratagene, La Jolla, CA). The parental pCS2+ vector was kindly provided by Sergei Tevosian (Dartmouth Medical School, Hanover, NH). Full-length mouse FOG-2 wt and mutants were also subcloned into the pEGFP-N2 vector. The fusion construct SUMO-1-FOG-2 mutant was synthesized by Genscript (Genscript, NJ) and included human SUMO-1 (1–97) and mouse FOG-2-4KR (1-1151) linked by two alanine residues. The constructs were cloned into the pcDNA3.1 vector. The FOG-2 deletion constructs FOG-2 509–1151, 729–1151 and 881–1092 were amplified by PCR and cloned into the pCS2+ vector. Murine GATA-4 was cloned into the Xho*I* and Bam*HI* sites of pCS2+. EGFP-SUMO-1 has been previously described [25] and was kindly provided by Hisato Saitoh (Picower Institute of Medical Research, New York, NY). The brain natriuretic peptide (BNP) reporter construct and pMT3-HA-SUMO-1 have been previously described [19], [26]. FLAG-SENP-1 (Addgene plasmid 17357) and FLAG-SENP-8 (Addgene plasmid 18066) were kindly provided by Edward Yeh (University of Texas, Houston, TX) [27], [28]. pCMV5-Myc-PIAS1, pCMV5-Myc-Miz1, pcDNA3-ARIP3 and pcDNA3-PIASy have been previously described [19].

### Cell Culture

Mouse myoblast C2C12 cells, African green monkey kidney fibroblasts (COS-7 cells) and HeLa cells were used for transfections. Cells were obtained from ATCC (Rockville, MD). Cells were cultured in Dulbecco’s Modified Eagle Medium (DMEM) (Invitrogen) supplemented with 10% dialyzed fetal bovine serum (Invitrogen) and maintained at 37°C in a humidified atmosphere of 5% CO_2_, 95% air. Neonatal rat cardiomyocytes were obtained from Lonza and cultured following the manufacturer’s instructions (Lonza, Waverly, VIC, Australia).

### Nuclear Localization, Transfections and Luciferase Assays

COS-7 were grown on coverslips and transiently transfected with 1–2 µg of GFP-FOG-2, GFP-FOG-2-4KR and FLAG-SENP1 expression vectors using Lipofectamine2000 following the manufacturer’s instructions (Invitrogen). Cells were fixed with 4% paraformaldehyde 48 hours after transfection, stained with PI (50 µg/ml) and analyzed with an Olympus confocal microscope (Olympus, Tokyo, Japan) at 600X magnification. Images were acquired using Olympus Fluoview software, version 4.3, FV300 (Olympus Optical Co. Ltd.). For protein expression, COS-7 cells were grown on 100 mm-diameter Petri dishes and transfected with 1–2 µg of FOG-2 and its derivatives, SUMO-1 or GFP-SUMO-1 expression vectors using Lipofectamine2000 following the manufacturer’s instructions (Invitrogen). HeLa cells used for luciferase assays were cultured in 6-well plates and were transfected with 300 ng of pGL3-Basic-BNP reporter [26], 300 ng of pCS2+GATA-4, 50 to 400 ng of pCS2+FOG-2, pCS2+FOG-2-4KR or SUMO-1-FOG-2-4KR, 50 to 300 ng of GFP-SUMO-1 and 500 ng of pFLAG-SENP-1 and pFLAG-SENP-8. The total amount of DNA was kept constant by adding empty pCS2+ vector. pRL-CMV was used as internal control (3 ng). The transfections were done using Lipofectamine2000 as previously mentioned. Cells were harvested 24 hours after transfection and the luciferase activity measured in a Turner Designs model TD 20/20 luminometer using the dual-luciferase reporter system (Promega). All data shown represent the results of three independent experiments. Cardiomyocytes were nucleofected using the 4D nucleofector (Solution P3, pulse DG-119, Lonza, Waverly, VIC, Australia) with 150 ng of pGL3-Basic-BNP reporter, 150 ng of pCS2+GATA-4, 50 to 200 ng of pCS2+FOG-2, pCS2+FOG-2-4KR or SUMO-1-FOG-2-4KR and 2 ng of pRL-CMV. Cells were harvested and the luciferase activity measured as described above.

### Antibody Staining

For nuclear localization studies the transfected cells were fixed in 2% paraformaldehyde, permeabilized in 100% methanol at –20°C, for 20 minutes, blocked with 5% skim milk in PBS and stained with anti-FLAG antibody (Sigma, 1∶800) for 45 minutes. Cells were washed three times with PBS and then were incubated for 45 minutes with the secondary antibody (anti-mouse Alexa Fluor 594, A-11020; Invitrogen, 1∶500 dilution). After washing, the coverslips were mounted on slides with DAPI mounting medium (Vectashield) and visualized using a Leica DM IRB inverted fluorescent microscope running Leica IM50, Version 1.20 software (Leica Microsystems, Heerbrugg, Switzerland).

### Western Blot Analysis

**Figure 1:**
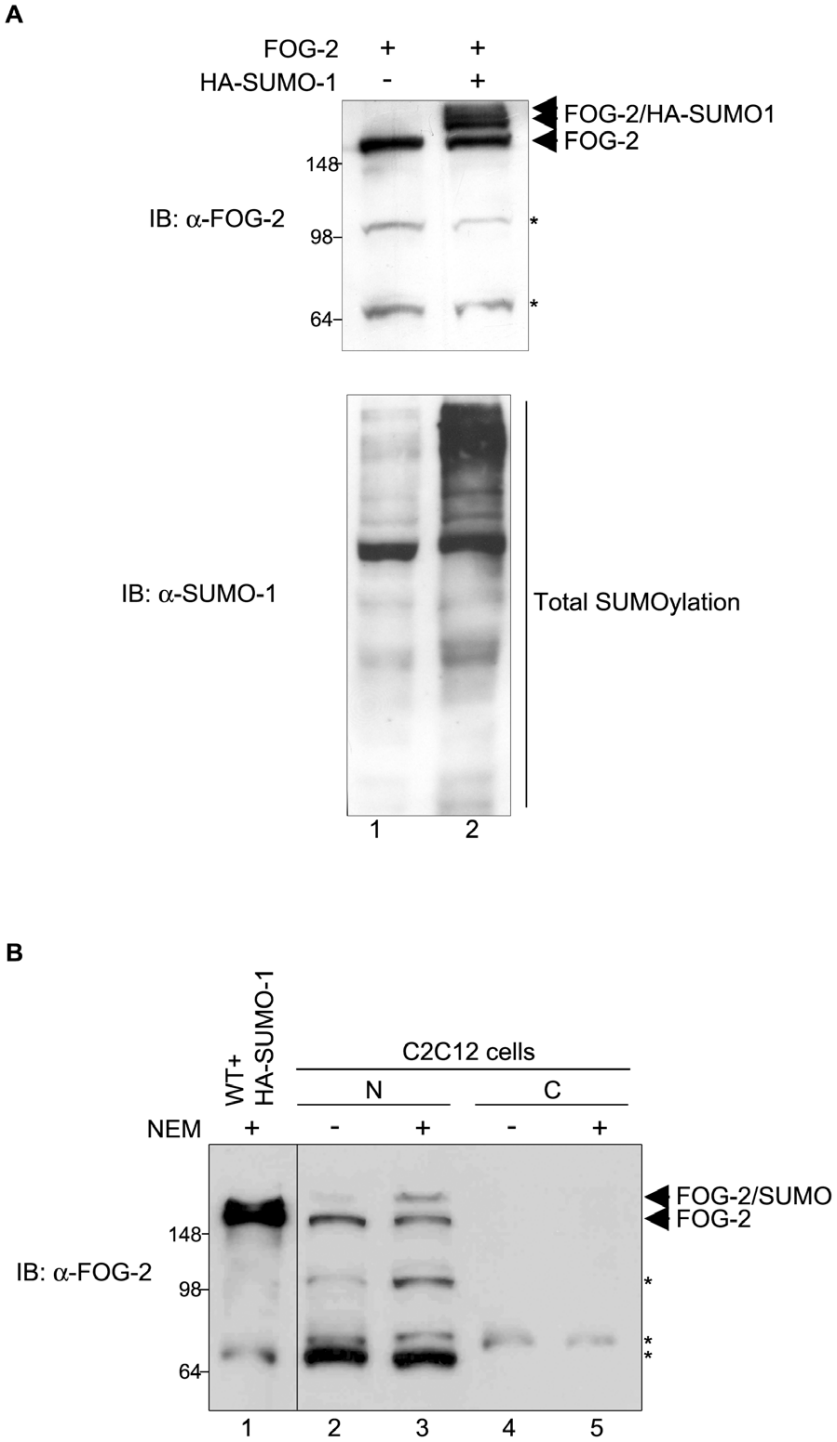
FOG-2 is SUMOylated in COS-7 and C2C12 myocytes. (A) COS-7 cells were transfected with expression vectors for murine FOG-2 (lane 1) or FOG-2 and HA-SUMO-1 (lane 2). Western blot was performed on nuclear extracts from the transfected cells using an anti-FOG-2 antibody. Co-expression of FOG-2 and HA-SUMO-1 resulted in the appearance of at least two slower migrating bands detected by the FOG-2 antibody (Upper panel, arrowheads). Expression of HA-SUMO-1 resulted in increased total SUMOylation (lower panel). SUMOylation in COS-7 cells was detected with an anti-SUMO-1 antibody in the absence (lane 1) or presence (lane 2) of co-transfected HA-SUMO-1 (B) Nuclear and cytoplasmic extracts from C2C12 myocytes were obtained in the absence (lanes 2 and 4) or presence (lanes 3 and 5) of the de-SUMOylation inhibitor NEM. FOG-2 was detected by the anti-FOG-2 antibody in the nuclear fraction (lanes 2 and 3). A slower migrating band appeared only when NEM was present, indicating that endogenous FOG-2 is modified by SUMO in these cells (lane 3). FOG-2 SUMOylated with HA-SUMO-1 in COS-7 cells is shown for comparison (lane 1). Asterisks indicate non-specific bands detected by the FOG-2 antibody. N, nuclear fraction; C, cytoplasmic fraction.

**Figure 2:**
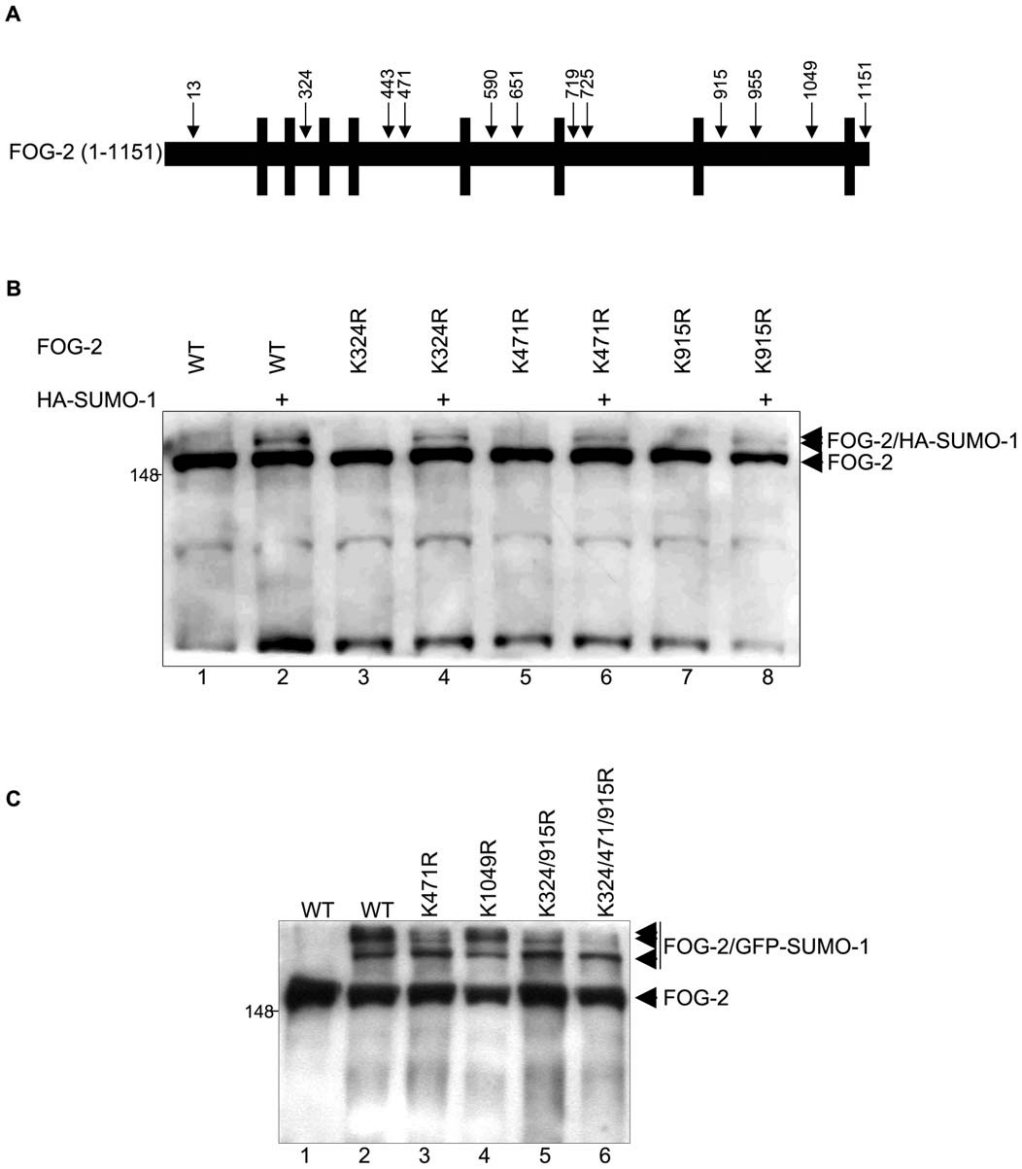
FOG-2 is SUMOylated at multiple sites. (A) Schematic representation of murine FOG-2. The position of lysine residues with a high probability of SUMOylation is indicated. Black vertical bars represent zinc fingers. (B) Nuclear extracts from COS-7 cells transfected with FOG-2 wt and the mutants indicated in the presence (+) or absence of HA-SUMO-1 were probed with an anti-FOG-2 antibody. Mutation of K324, 471 and 915 reduced but did not eliminate the FOG-2 SUMOylation. (C) An expression vector for GFP-SUMO-1 was cotransfected with FOG-2 wt and the indicated single, double and triple mutants (lanes 2 to 6). FOG-2 and slower migrating species, representing FOG-2 SUMOylated by GFP-SUMO-1, are indicated by arrowheads. Expression of the triple mutant (K324/471/915R, lane 6) abolished almost all SUMOylation except one band.

**Figure 3:**
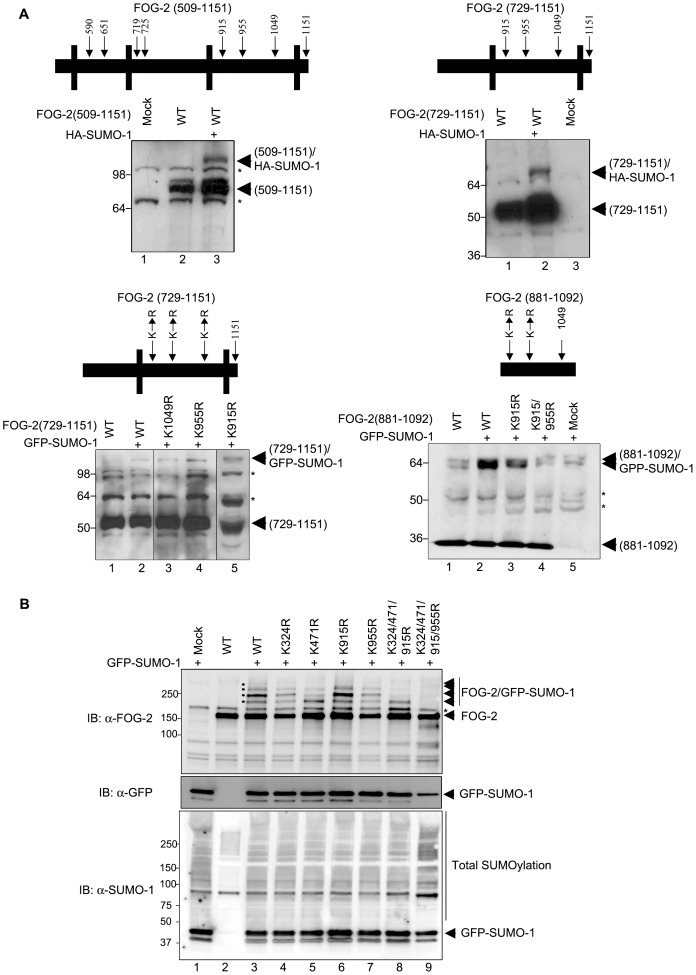
Mapping of FOG-2 SUMOylation. (A) A series of FOG-2 deletion fragments (509-1121, 729-1151, 881-1092) were transfected into COS-7 cells together with expression vectors for HA-SUMO-1 or GFP-SUMO-1 as indicated in the figure. Mutations in fragments 279-1151 and 881-1092 are indicated in the figure. All FOG-2 fragments were SUMOylated. Mutation of K915 and K955 in the 881-1092 fragment caused the disappearance of the previous SUMOylation band (lane 4, lower panel), indicating that K955 is a site for SUMO modification in FOG-2. (B) FOG-2 wt and single, triple and quadruple mutants were co-expressed with GFP-SUMO-1 (lanes 3 to 9). The SUMOylated FOG-2 species are indicated by black dots in the blot and by arrowheads (upper panel). Mutation of K955 (lane 7) abolished the fourth SUMOylation band, indicating that, apart from K955, FOG-2 possesses 3 additional SUMO acceptor sites. Mutation of the other 3 sites (K324/471/915) results in a single SUMOylation band corresponding to K955 (lane 8). Mutation of K324/471/915/955R led to the abolition of FOG-2 SUMOylation (lane 9). The expression of GFP-SUMO-1 and the total SUMOylation levels in the cell extracts are shown in the middle and lower panels, respectively. IB, immunoblot.

To detect SUMOylated FOG-2 and its derivatives, Western blot analyses were carried out in the presence of the de-SUMOylation inhibitor N-ethylmaleimide (NEM) at a final concentration of 25 mM essentially as previously described [19]. For transfected COS-7 cells, nuclear extracts [19] or whole cell lysates were analyzed. Membranes were probed with the following primary antibodies (Ab): rabbit anti-FOG-2 Ab (sc-10755; Santa Cruz; 1∶200), rabbit anti-GATA-4 (sc-9053; Santa Cruz; 1∶500) mouse anti-β-actin (clone AC-15, Sigma, 1∶5000), rabbit anti-GFP (Ab290, Abcam; 1∶5000) and mouse anti-SUMO-1 (1∶300). The secondary antibodies used were anti-rabbit-HRP Ab (sc-2054; Santa Cruz; 1∶5000 dilution) and anti-mouse HRP Ab (P0260; DAKO, Denmark; 1∶3000 dilution). Signals were detected using Western Lightning Chemiluminescence Reagent Plus (Perkin Elmer, Wellesley, MA) and CL-Xposure Film (Quantum Scientific, QLD, Australia) (for Figs. 1 to 3A and Fig. S1) or with the ImageQuant LAS 4000 imager (GE Healthcare).

### Immunoprecipitation

Immunoprecipitations were carried out using anti-GFP magnetic beads (µMACS™ Epitope Tag Protein Isolation Kit, Miltenyi Biotec, Germany) following the manufacturer’s instructions. Briefly, ∼ 2×10^6^ cells were washed with PBS and then lysed in 1 ml of lysis buffer (150 mM NaCl, 1% Triton X-100, 50 mM Tris, pH 8.0, 25 mM NEM). Then 50 µl of anti-GFP microbeads was added and the mixture incubated on ice of 30 min. The bound proteins were separated using a µ column and a µMACS magnetic separator. After washing 2×200 µl with Buffer 1 (150 mM NaCl, 1% igepal CA-630, 0.5% sodium deoxycholate, 0.1% SDS, 50 mM Tris pH 8.0, 25 mM NEM) and once with 100 µl of Buffer 2 (20 mM Tris, pH 7.5, 25 mM NEM), the proteins were eluted with 70 µl of boiling elution buffer (50 mM Tris, pH 6.8, 50 mM DTT, 1% SDS, 1 mM EDTA, 0.005% bromophenol blue and 10% glycerol). The proteins were resolved by SDS-PAGE and detected by Western blotting.

### Statistics

Data represent the mean and corresponding standard deviation. The probability values obtained by unpaired, 2-tailed Student’s t-tests were considered significant if 0.01<p<0.05 (represented by *) and very significant if p<0.01 (represented by **).

## Results

### FOG-2 is a Target for SUMOylation

Inspection of the mouse FOG-2 sequence with the SUMOsp package [29] found 12 lysine residues that were potential SUMOylation sites (Table 1). These putative SUMOylated amino acids are distributed along the molecule but are not found within zinc finger domains. To determine if FOG-2 is a substrate for SUMO modification, COS-7 cells were transfected with a plasmid encoding murine FOG-2 in the presence or absence of a SUMO-1 expression vector (it is convenient to analyze the SUMOylation of proteins which might possess multiple SUMOylation sites with SUMO-1 since it does not form poly-SUMO chains). Nuclear extracts were subjected to Western blot analysis with an anti-FOG-2 antibody. FOG-2 was readily detected at an apparent molecular mass of approximately 180 kDa (Fig. 1A). FOG-2 is typically observed at an apparent molecular mass higher than the predicted 127 kDa in COS-7, HeLa, 293 Cells and in cell free transcription/translation systems (J. Perdomo, unpublished). Svensson et al [30] also observed FOG-2 at a higher molecular mass in COS-7 cells and in an in vitro transcription/translation system. Higher molecular mass species were detected with the anti-FOG-2 antibody only when the SUMO-1 expression vector was present (Fig. 1A, arrowheads) indicating that FOG-2 can be modified by SUMO-1 when both proteins are co-expressed in COS-7 cells.

**Table 1:** Predicted SUMOylation sites of murine FOG-2 using the SUMOsp program.

*Position*	*Peptide*	*Score*	*Type*
13	RQI**K**RPL	2.368	Non-consensus
324	SGV**K**MEE	2.796	Ψ-K-X-E
443	KCE**K**KTQ	2.412	Non-consensus
471	TKI**K**SEP	6.005	Ψ-K-X-E
590	VSE**K**MPE	2.294	Non-consensus
651	TQV**K**KLP	2.353	Non-consensus
719	PPL**K**RSA	2.632	Non-consensus
725	ASN**K**VPA	2.353	Non-consensus
915	NMI**K**CEK	1.839	Ψ-K-X-E
955	IAT**K**EEN	2.544	Non-consensus
1049	GGL**K**QDE	2.574	Non-consensus
1151	EHVK***	3.294	Non-consensus

To ascertain if endogenous FOG-2 was modified by SUMO, nuclear and cytoplasmic extracts were obtained from C2C12 myoblasts in the presence or absence of the SUMO isopeptidase inhibitor NEM, which prevents deSUMOylation. A slower migrating band was detected in the nuclear fraction by the FOG-2 antibody only in the presence of NEM (Fig. 1B), indicating that endogenous FOG-2 is modified by SUMO in C2C12 cells.

### FOG-2 is SUMOylated at Lysines 324, 471, 915 and 955

Lysine residues with a high probability of SUMOylation are shown schematically in Fig. 2A. Three of these lysines (324, 471 and 915), fall within canonical SUMOylation sites, while the other predicted residues are part of non-consensus sequences (Table 1). The putative SUMOylated lysines within the consensus sequences were mutated to arginine and vectors encoding these constructs were transfected into COS-7 cells in the presence or absence of HA-SUMO-1. Fig. 2B shows that both wild-type and the mutants K324R, K471R or K915R were SUMOylated by HA-SUMO-1, suggesting that there may be more than one acceptor site in FOG-2.

It is apparent in Fig. 1A that FOG-2 is being modified by more than one SUMO-1 moiety (Fig. 1A, arrowheads). However, the high molecular mass of FOG-2 precluded unambiguous separation of the SUMOylated species as SUMO-1 increases the apparent molecular mass of modified proteins by only approximately 20 kDa. For this reason, COS-7 cells were co-transfected with expression vectors for FOG-2 and a GFP-SUMO-1 fusion that increases the size of the SUMO moiety to approximately 50 kDa. At least 3 slower migrating species were observed (Fig. 2C, lane 2, arrowheads) indicating that more than two lysine residues in FOG-2 could be targeted by SUMO-1. A number of single and combination mutants were generated and then expressed in COS-7 cells and analyzed by Western blot. Fig. 2C, lanes 3–6, shows a selection of these mutants. Combinations of double and triple mutants revealed that all SUMOylation bands, except one, were abolished when lysine residues 324, 471 and 915 were mutated to arginine (Fig. 2C, lane 6).

Mutation of several other residues that also had a high theoretical probability of being SUMOylated such as K729 and K1049 in conjunction with residues 324, 471 and 915 did not prevent the appearance of a single SUMOylation band (data not shown). To define the region of the last SUMOylation site of FOG-2, a series of deletion mutants was created and then subjected to SUMOylation in COS-7 cells (Fig. 3A). Fragments 509–1151 and 729–1151 were SUMOylated at apparently a single site (Fig. 3A, FOG-2(509–1151), lane 3 and FOG-2(729–1151), lane 2). Single point mutations in the 729–1151 fragment did not completely abrogate SUMO modification (Fig. 3A, FOG-2(729–1151)KR mutants, lanes 3–5). Additional mutations in the fragment containing amino acids 881–1092 of FOG-2 identified K955 as a SUMO-acceptor residue, and mutation of this amino acid, in combination with K915, resulted in the complete elimination of SUMOylation of this FOG-2 fragment (Fig. 3A, FOG-2(881–1092), lane 4). The validity of this result was then confirmed in the context of the full-length protein. Fig. 3B shows that the quadruple mutant (from now on termed FOG-2-4KR) is not modified by SUMO-1 (Fig. 3B, lane 9, upper panel). Thus K324, K471, K915 and K955 are the only SUMO acceptor sites in mouse FOG-2.

**Figure 4:**
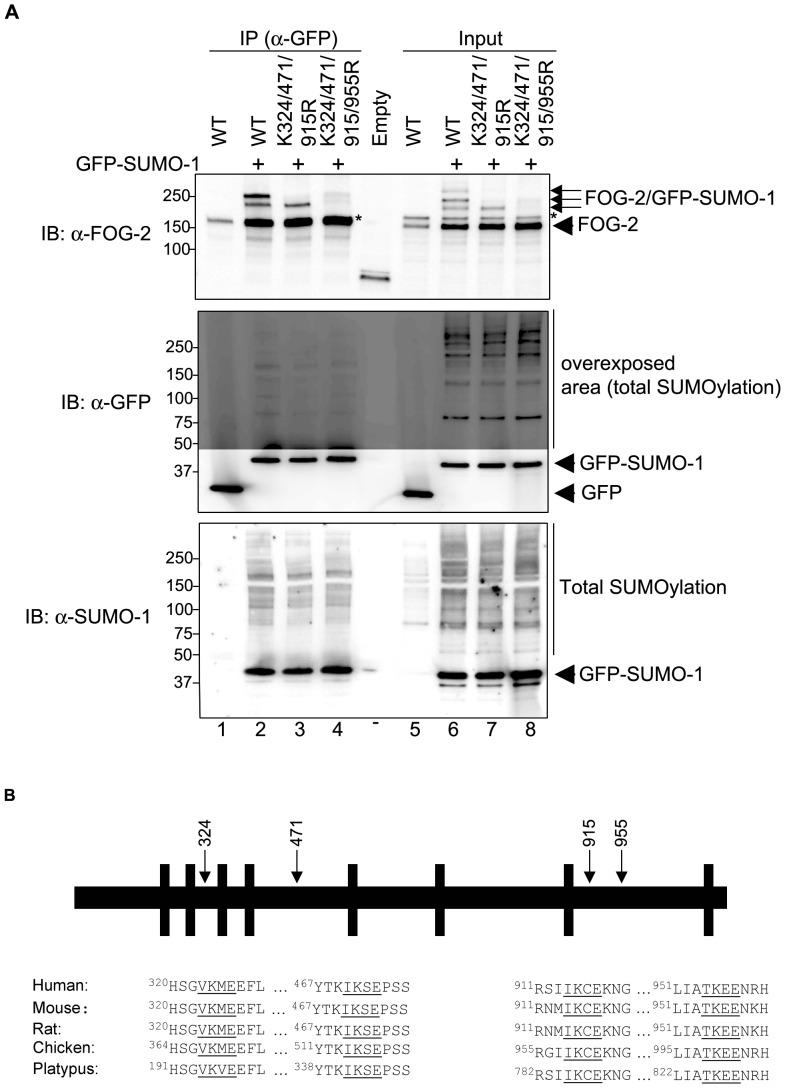
Confirmation of FOG-2 SUMOylation sites. (A) COS-7 cells were co-transfected with GFP or GFP-SUMO-1 and either wt FOG-2, FOG-2 triple SUMOylation site mutant (K324/471/915R) or FOG-2 quadruple mutant (K324/471/915/955R). Immuno-precipitation experiments were performed in cell extracts using magnetic beads coated with an anti-GFP antibody. Immuno-precipitated complexes and cell lysates (input) were resolved by SDS-PAGE and blotted with anti-FOG-2, anti-GFP or anti-SUMO-1 antibodies. Immuno-precipitated FOG-2 SUMOylated by GFP-SUMO-1 is observed in lane 2, upper panel. A single SUMOylated band is seen in the triple mutant (lane 3, upper panel), while the FOG-2 K324/471/915/955R mutant is not SUMOylated (lane 4, upper panel). Protein input (5%) in shown in lanes 5 to 8. Expression of GFP alone (lanes 1 and 5), GFP-SUMO-1 (lanes 2–4 and 6–8) and total SUMOylation are shown in the middle and lower panels. Asterisks indicate non-specific bands detected by the FOG-2 antibody (this non-specific band was enriched by the immuno-precipitation - upper panel, lanes 2 to 4 - indicating that this cross-reacting species is also a SUMOylated protein). (B) The alignment of FOG-2 sequences from human to platypus (Ornithorhynchus anatinus) shows conservation of the four SUMOylation sites. Asterisks indicate non-specific bands detected by the FOG-2 antibody. IB, immunoblot; IP, immuno-precipitation.

The mapping of the FOG-2 SUMOylation sites was confirmed by immunoprecipitation of SUMOylated FOG-2 wt, K324/471/915R and 4KR. Fig. 4A shows that at least two SUMOylated bands are pulled down by the anti-GFP antibody when FOG-2 wt is SUMOylated by GFP-SUMO-1 (Fig. 4A, lane 2, upper panel). The anti-GFP antibody also precipitated a single SUMOylated band when the K324/471/915R mutant molecule was used as a substrate (Fig. 4A, lane 3, upper panel). In contrast, no SUMOylated FOG-2 was immunoprecipitated when the K324/471/915/955R mutant was co-expressed with GFP-SUMO-1 (Fig. 4A, lane 4, upper panel). Collectively, these experiments show that FOG-2 is targeted for SUMO modification at K324, 471, 915 and 955. The four SUMOylation sites identified in murine FOG-2 show strong conservation across the species examined (Fig. 4B), which would suggest preserved biological functionality.

### SUMOylation does not Affect the Cellular Distribution of FOG-2

**Figure 5:**
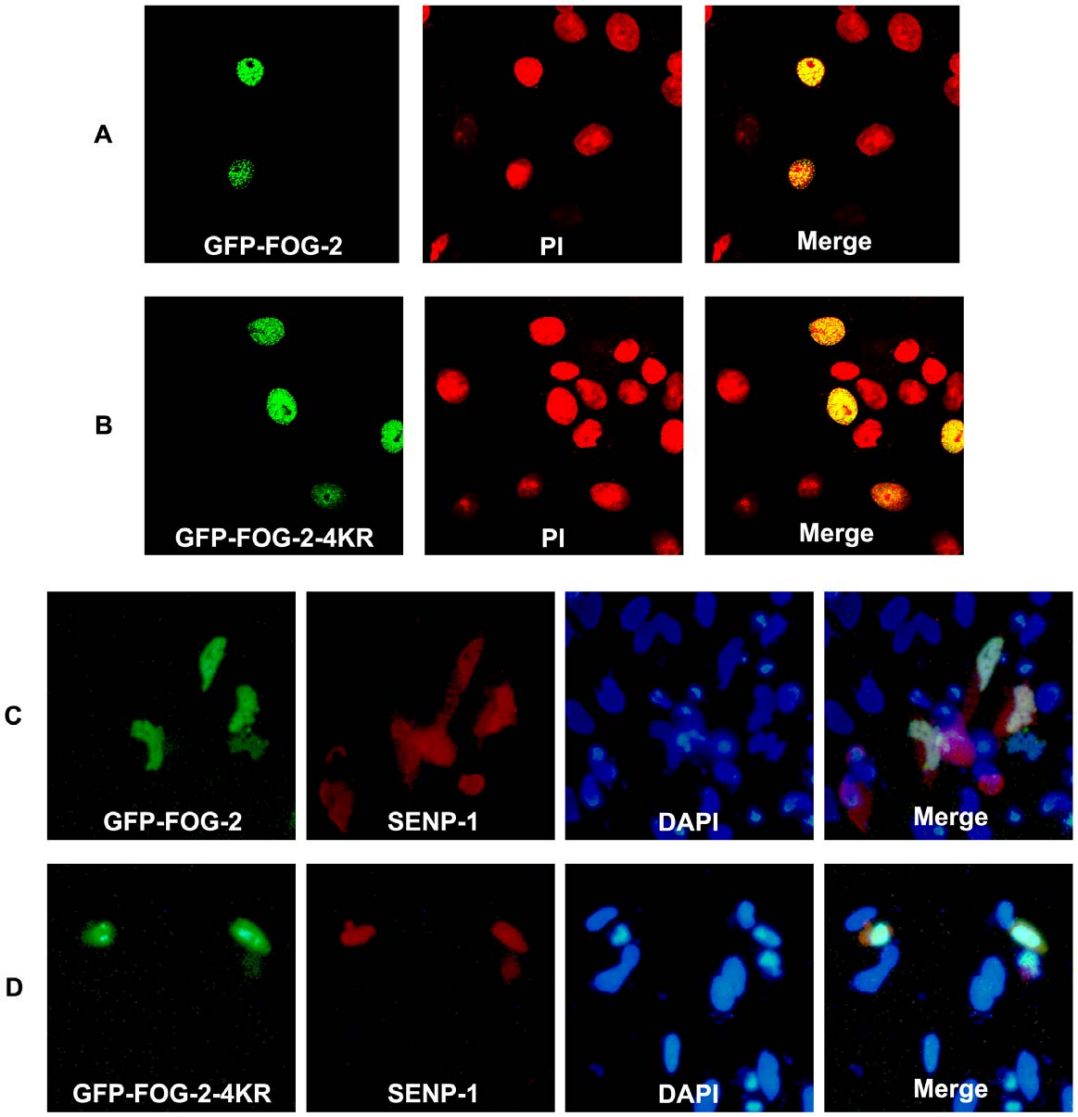
The sub-cellular localization of FOG-2 is not affected by SUMOylation. COS-7 cells were transfected with (A) GFP-FOG-2 or (B) GFP-FOG-2-4KR fusion proteins. The cell nuclei were stained with PI (red). There was no detectable difference in the sub-cellular or sub-nuclear distribution of wt and mutant FOG-2. COS-7 cells were co-transfected with FLAG-SENP-1 and (C) GFP-FOG-2 or (D) GFP-FOG-2-4KR fusion proteins. FLAG-SENP-1 was detected with anti-FLAG antibody and with anti mouse IgG-Alexa-594 (red). Cell nuclei were stained with DAPI (blue). The presence of FLAG-SENP-1 did not affect the sub-cellular distribution of FOG-2.

For several proteins the connection between SUMO modification and nuclear translocation has already been established (for review see [31]). We expressed GFP-FOG-2 fusion proteins in COS-7 cells and asked whether intact SUMOylation sites were required for nuclear localization. As expected, wt FOG-2 was found exclusively in the nucleus of more than 95% of the transfected cells examined (Fig. 5A). When the SUMOylation deficient mutant GFP-FOG-2-4KR was expressed in these cells it also localized to the nucleus (Fig. 5B), indicating that SUMOylation plays no role in the cellular distribution of FOG-2 in COS-7 cells. Similar results were obtained when GFP-FOG-2 and GFP-FPG-2-4KR were expressed in HeLa cells (Fig. S1B). In addition, co-expression of FOG-2 with the de-SUMOylating enzyme SENP-1 (this enzyme removes SUMO-1 from FOG-2, Fig. 7) did not alter the sub-cellular localization of FOG-2 (Fig. 5C), thus confirming that SUMOylation is not necessary for FOG-2′s nuclear targeting.

### Lack of SUMOylation Potentiates FOG-2′s Repression Capacity

The transcriptional activity of FOG proteins was demonstrated previously in transient transfection assays in heterologous cells [11], [12], [32]. Therefore, it was pertinent to determine if SUMO modification was required for the biological activity of FOG-2. These experiments were first conducted in HeLa cells using the luciferase reporter gene under the control of the GATA-4-responsive cardiac BNP promoter.

**Figure 6:**
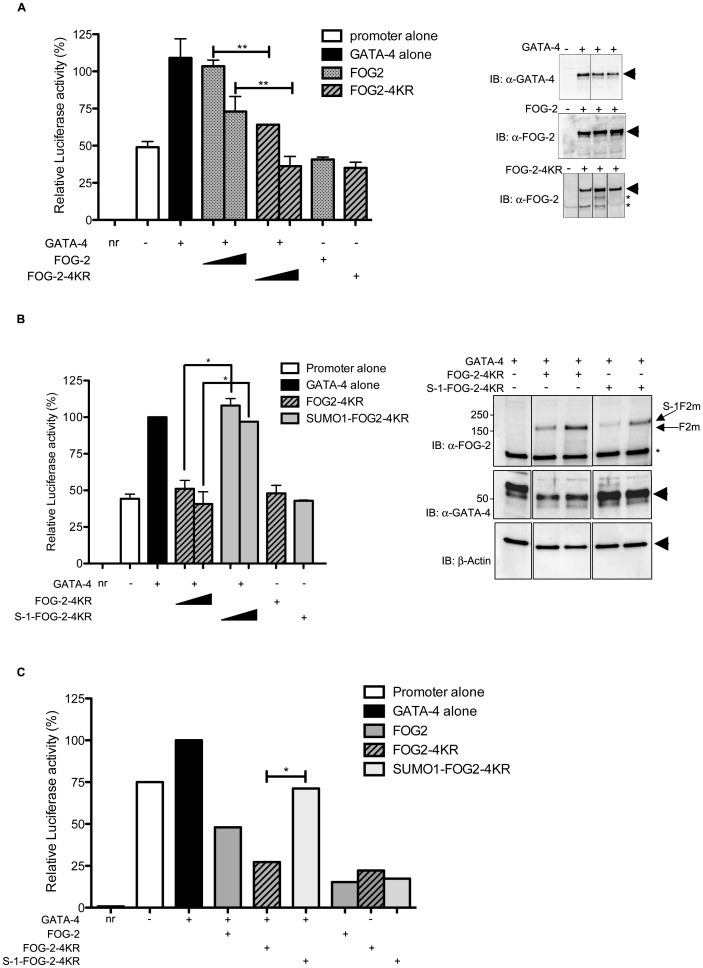
The repression activity of FOG-2 is hindered by SUMOylation in HeLa cells and in rat cardiomyocytes. HeLa cells were co-transfected with a reporter construct containing the luciferase gene under the control of the BNP promoter and the indicated vectors. Dual luciferase assays were performed 24 h post-transfection. (A) The BNP promoter was activated by GATA-4 and, as expected, wt FOG-2 repressed this activation. Expression of the SUMOylation mutant molecule (FOG-2-4KR) resulted in significantly stronger repression activity. The immunoblots represent total cell lysates from the transfected cells probed with the antibodies indicated in the Figure. (B) The BNP promoter was activated by GATA-4 and this activation was strongly repressed by FOG-2-4KR. The constitutively SUMOylated fusion protein SUMO-1-FOG-2-4KR failed to repress GATA-4-mediated activation. The immunoblots represent total cell lysates from the transfected cells probed with the antibodies indicated in the Figure. Data represent the mean ± SD from 3 independent experiments (C) Neonatal rat cardiomyocytes were nucleofected with the vectors indicated in the Figure. In agreement with the findings in *A* and *B*, FOG-2-4KR showed stronger repression activity than the wt, while the SUMO-1-FOG-2-4KR fusion demonstrated significantly reduced repression capacity. Of note, co-expression of GATA-4 in cardiomyocytes did not activate the BNP promoter significantly most likely due to quenching by the presence of endogenous GATA-4. In addition, the repression exerted by FOG-2 and FOG-2-4KR reduced luciferase activity to levels lower than the reporter itself, again suggesting that the promoter alone was activated by endogenous GATA-4 and that FOG-2 repressed this activity. Data represent the arithmetic mean from 2 independent experiments. IB, immunoblot; ns, not significant; nr, no reporter; S-1, SUMO-1; F2m, FOG-2-4KR; S-1F2m, SUMO-1-FOG-2-4KR.

As reported previously [30], GATA-4 increased the activity of the reporter gene significantly and this activation was repressed by co-expression of increasing amounts of wild-type FOG-2 (Fig. 6A). To investigate whether SUMOylation was important in FOG-2 repression we also examined the effect of FOG-2-4KR in the same transcription assay. Fig. 6A shows that the non-SUMOylated FOG-2 molecule was a more potent transcriptional repressor, indicating that lack of SUMOylation enhances FOG-2-mediated transcriptional repression.

Having shown that mutation of the SUMO acceptor lysines in FOG-2 led to enhanced repression capacity, we wished to corroborate the observations using an artificially SUMOylated molecule. It has been shown that mimicking SUMOylation by fusing SUMO to a substrate can recapitulate to a large extent the effects of SUMO modification at the natural target sites [33]. To this end, we fused SUMO-1 at the N terminus of mutant FOG-2 (SUMO-1-FOG-2-4KR) and tested the transcriptional activity of this chimeric construct. Fig. 6B shows that expression of SUMO-1-FOG-2-4KR abolished the capacity of FOG-2-4KR to repress GATA-4-mediated transcription, thus implicating SUMOylation in a mechanism that leads FOG-2 to alternate between a repressive and a more permissive transcriptional status. Even though SUMO fusion proteins are artificial and probably exhibit an aberrant level of SUMOylation (the fusion protein is constantly SUMOylated), the fact that SUMO-1-FOG-2-4KR reversed the repression activity of FOG-2-4KR strongly implies that SUMOylation attenuates FOG-2-mediated repression.

We next examined whether SUMOylation is relevant for the transcriptional activity of FOG-2 in cardiac cells. Amaxa® nucleofection technology was used to co-transfect the expression vectors indicated in Fig. 6C into neonatal rat cardiomyocytes. The transfection efficiency was determined visually by co-transfection of a GFP expression vector. The data shown in Fig. 6C substantiates the observations in HeLa cells, with FOG-2-4KR demonstrating augmented repression capacity and the SUMO-1-FOG-2-4KR chimera neutralizing the repressive competence.

**Figure 7:**
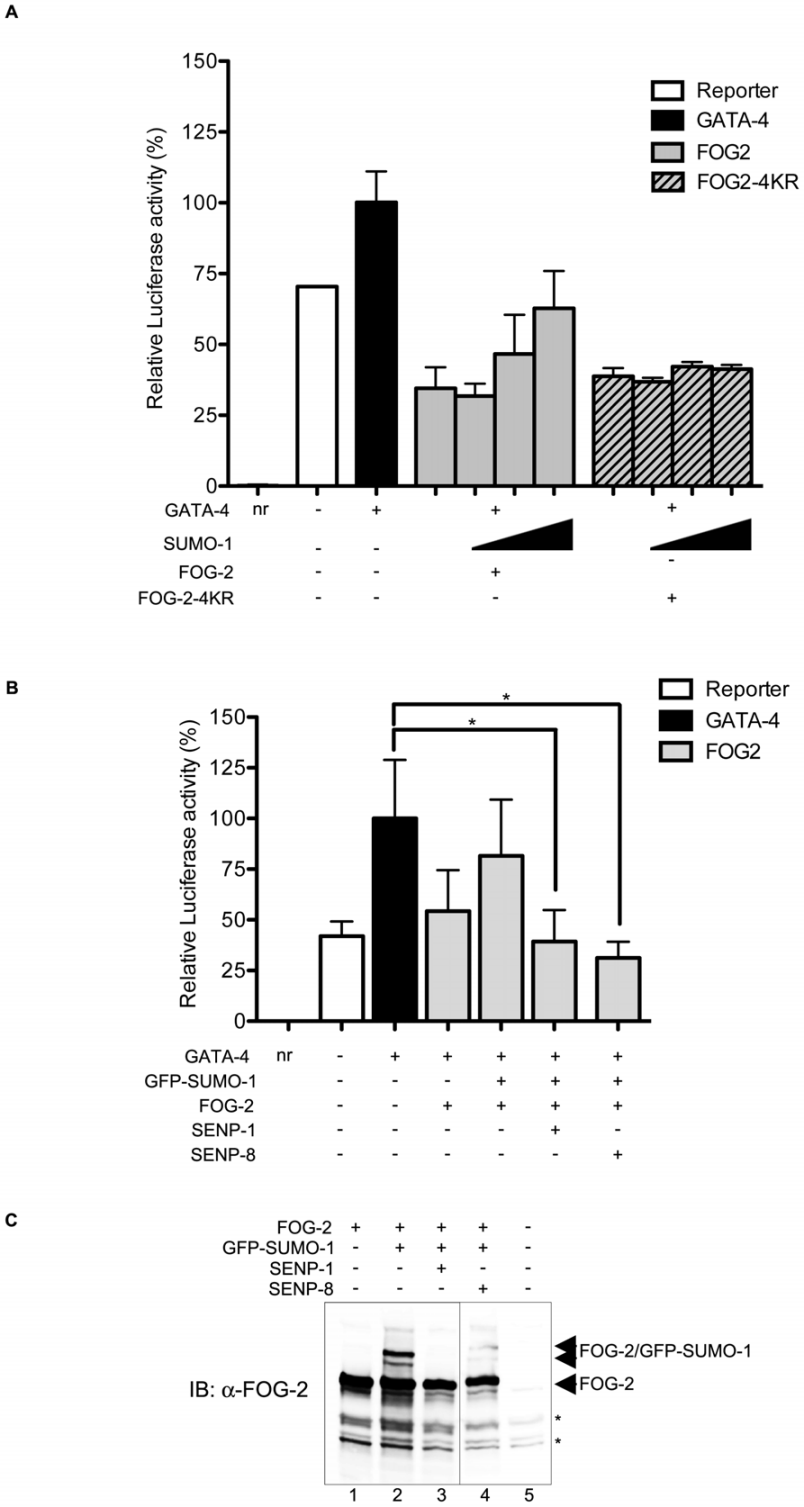
FOG-2 SUMOylation and de-SUMOylation have antagonistic effects on its repression activity. (A) HeLa cells were co-transfected with the BNP-Luciferase reporter and wt FOG-2 or FOG-2-4KR together with increasing amounts of SUMO-1. Increasing expression of SUMO-1 resulted in reduced repression by FOG-2. Expression of SUMO-1 did not affect the repression capacity of the non-SUMOylatable 4KR mutant. (B) HeLa cells were co-transfected with the BNP-Luciferase reporter and wt FOG-2 together with SUMO-1 and FLAG-SENP-1 as indicated in the Figure. As shown in *A,* SUMOylation of FOG-2 by GFP-SUMO-1 reduced its repression activity. Conversely, de-SUMOylation by SENP-1 or SENP-8 increased FOG-2′s repression capacity. (C) Western blot showing FOG-2 de-SUMOylation by SENP-1 and SENP-8 from an experiment run in parallel (note that SUMOylation is not observed in the extracts used for luciferase assays because the inhibitor NEM is not included in the luciferase lysis buffer). Data represent the mean ± SD from 2 independent experiments. Asterisks indicate non-specific bands detected by the FOG-2 antibody. IB, immunoblot; nr, no reporter.

Moreover, co-expression of increasing amounts of SUMO-1 in HeLa cells reduced the repression activity of wt FOG-2 but not that of FOG-2-4KR (Fig. 7A). As anticipated from their function, co-expression of the SUMO-specific de-SUMOylating enzymes SENP-1 and SENP-8 resulted in the abrogation of FOG-2 SUMOylation (Fig. 7C, lanes 3 and 4). Notably, co-expression of both SENP-1 and SENP-8 also led to a significant increase in FOG-2′s repression capacity in the presence of SUMO-1 (Fig. 7B). Altogether, the data imply that absence of SUMOylation renders FOG-2 a more effective transcriptional repressor.

### GATA-4 Regulates FOG-2 SUMOylation

**Figure 8:**
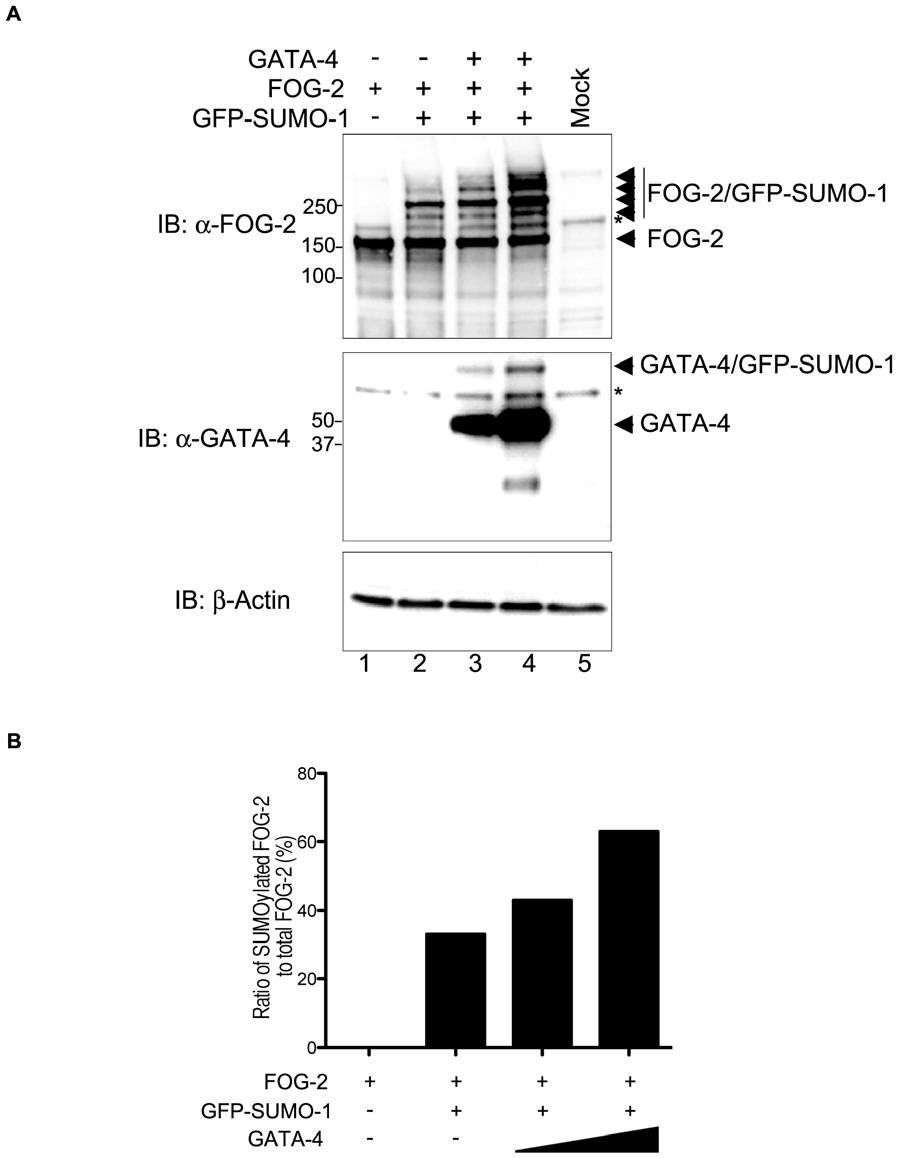
GATA-4 enhances FOG-2 SUMOylation. (A) COS-7 cells were transfected with constructs containing FOG-2, GFP-SUMO-1 and GATA-4 as indicated in the figure. Cells were boiled directly in Laemmli buffer, run for Western blotting and probed with the indicated antibodies. (B) The increase in FOG-2 SUMOylation was quantitated by densitometry using ImageQuant TL 1D, version 7.0 (GE Healthcare). The graph shows the ratio of total SUMOylated FOG-2 to total FOG-2 (percentage). Asterisks indicate non-specific bands detected by the FOG-2 antibody. IB, immunoblot.

SUMO E3 ligases such as PIAS1 and PIAS2 are expressed in the heart [34] and GATA-4 SUMOylation is regulated by PIAS1 [35], [36]. Nevertheless, co-expression of FOG-2 with SUMO-1 and the E3 ligases PIAS1, PIAS2 (Miz1), PIAS3 (ARIP-3) and PIAS4 (PIASy) did not enhance FOG-2 SUMOylation (Fig. S1A). In addition, co-expression of the SUMO E2 ligase Ubc9, did not increase FOG-2 SUMOylation, suggesting that this enzyme is not a limiting factor in COS-7 cells (Fig. S1A, lanes 2 and 7). Nonetheless, we noticed that co-expression of FOG-2 and GATA-4 led to stronger FOG-2 SUMO modification. As seen in Fig. 8, co-expression of increasing amounts of GATA-4 resulted in a corresponding increase in FOG-2 SUMOylation (Fig. 8A, lanes 2 to 4 and Fig. 8B). This is reminiscent of the increase in FOG-1 SUMOylation seen in the presence of GATA-1 or GATA-2 ( [22] and our unpublished observations). Thus, the presence of GATA-4 favours FOG-2 SUMO modification and may represent a mechanism by which GATA factors may modulate FOG-2′s activity.

### The FOG-2/GATA-4 Interaction is Enhanced in the Absence of SUMOylation

**Figure 9:**
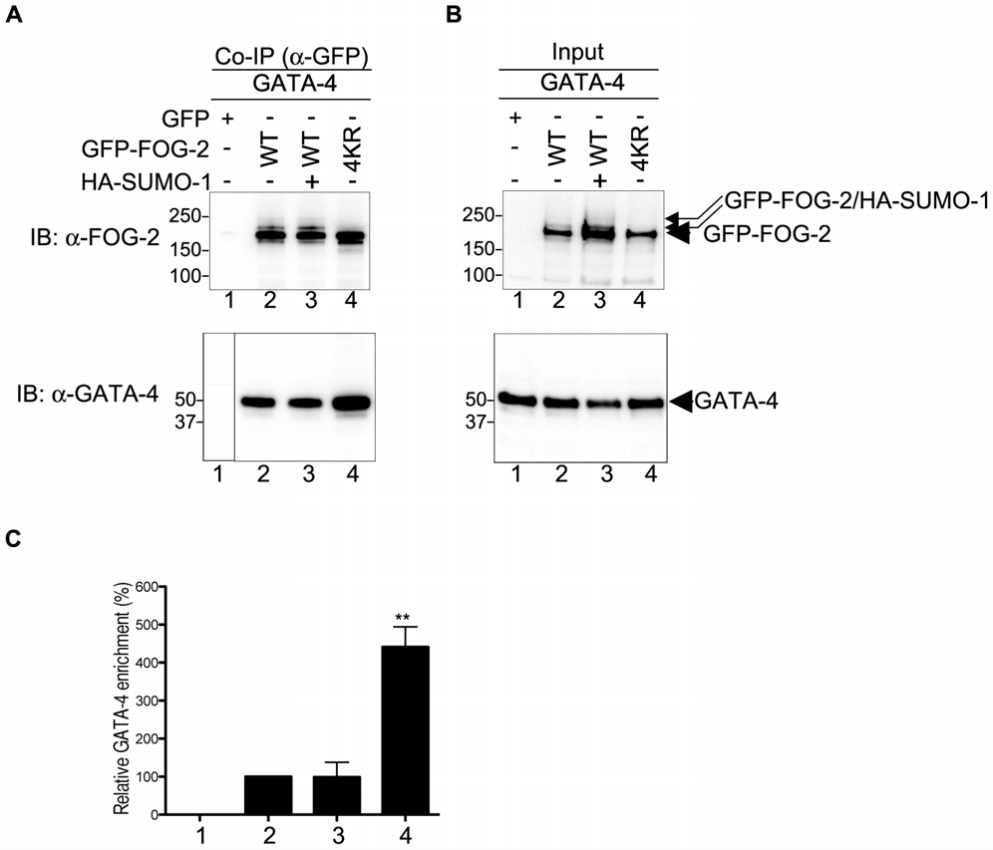
Lack of SUMOylation increases the protein-protein interaction between FOG-2 and GATA-4. COS-7 cells were transfected with constructs containing GFP alone, GFP-FOG-2 wt and 4KR mutant, HA-SUMO-1 and GATA-4 as indicated in the figure. Cell lysates were obtained in the presence of NEM. (A) Immuno-precipitation experiments were performed in cell extracts using magnetic beads coated with an anti-GFP antibody. Immuno-precipitated complexes were resolved by SDS-PAGE and blotted with anti-FOG-2 or anti-GATA-4 antibodies. (B) Cell lysates (5% input) were resolved by SDS-PAGE and blotted with anti-FOG-2 or anti-GATA-4 antibodies. Note that FOG-2 is SUMOylated by endogenous SUMO when GATA-4 is co-expressed (lane 2, upper panels). (C) The immuno-precipitation was repeated and GATA-4 was quantitated by densitometry using ImageQuant TL 1D, version 7.0 (GE Healthcare). The graph shows GATA-4 enrichment relative to immuno-precipitated FOG-2 (percentage).

The physical interaction between FOG-2 and GATA-4 is well established [30] and we sought to ascertain whether SUMO modification of FOG-2 altered this association. Immuno-precipitation of GFP-FOG-2 with anti-GFP magnetic beads, in the presence and absence of co-expressed HA-SUMO-1, resulted in co-precipitation of equivalent amounts of GATA-4 as assessed by the anti-GATA-4 antibody (Fig. 9A, lanes 2 and 3 and Fig. 9C, bars 2 and 3). No GATA-4 was detected in the GFP control (Fig. 9A, lane 1) (Of note, the immuno-precipitated GFP-FOG-2 was SUMOylated even in the absence of co-expressed HA-SUMO-1 due to the presence of co-expressed GATA-4). In contrast, the non-SUMOylated FOG-2-4KR co-precipitated an increased level of GATA-4 (Fig. 9A, lane 4 and Fig. 9C, bar 4). The experiment was repeated and comparable results were obtained, with a more than 3-fold relative increase in co-precipitated GATA-4 (p<0.01). Therefore, an increase in the FOG-2/GATA-4 association in the absence of FOG-2 SUMOylation is likely to be responsible for the augmented repression activity of FOG-2-4KR seen in the transcription assays reported here.

## Discussion

SUMO modification is a post-translational process regulates the biological activity of many proteins. The experiments presented in this study demonstrate that SUMOylation is a key factor in the biological function of the transcriptional co-regulator FOG-2. Specifically we show that: 1) FOG-2 undergoes SUMO modification and mutation of four specific lysines is sufficient to abrogate SUMOylation; 2) SUMOylation is not required for the nuclear distribution of FOG-2; 3) lack of SUMOylation switches FOG-2 into a more potent transcriptional repressor; and 4) there is a correlation between the FOG-2/GATA-4 interaction and SUMO modification.

Systematic mutation of putative SUMOylation sites in FOG-2 (Table 1) led to the identification of the first three SUMO acceptor lysines (K324, K471 and K915). These residues lie within the characteristic SUMO consensus sequence ψKXE, where the amino acid preceding the target lysine is large and hydrophobic, most commonly a valine, leucine or isoleucine. A fourth SUMOylation site (K955) was found within the less frequent TKEE sequence, where a hydrophilic residue, namely a threonine, precedes the target lysine. The TKXE consensus, though uncommon, has also been reported for TIF1β, p45-NF-E2 and TEL/ETV6 [37], [38], [39]. The four SUMOylation sites identified in murine FOG-2 are conserved across the species examined (Fig. 4B) suggesting functional conservation.

Most substrates contain only one or two SUMO acceptor residues [40]. There are some factors, however, with multiple SUMOylation sites; these include PML, GRIP1 and ELK-1 with three SUMOylation sites each [39], [41], [42] and TIF1β which is modified by SUMO at six positions [37]. Moreover, there appears to be preferential modification of certain residues, for instance BKLF is modified at one major and one minor site [19] while TIF1β contains three major and three minor SUMOylation sites [37]. In FOG-2, K471 and K955 are modified strongly by SUMO-1 while K324 and K915 are SUMOylated to a lesser extent (Fig. 2 and 3 and data not shown). SUMOylation of endogenous FOG-2 in C2C12 cells revealed only one main SUMOylated species. However, detection of all endogenous SUMOylated species is not always feasible due to the small amount of SUMO-conjugated proteins usually found in cells [43].

Although the role of SUMOylation in nuclear targeting has been established for some proteins [31], the nuclear localization of many other proteins is unaffected by SUMOylation [22], [34]. Our data show that nuclear targeting of FOG-2 in COS-7 and HeLa cells does not depend on the presence of intact SUMOylation sites, indicating that for FOG-2 SUMO modification is dispensable for nuclear transport. Nevertheless, SUMOylation was found to be functionally required for the transcriptional activity of FOG-2. Mutations that abolished SUMOylation, or de-SUMOylation by SUMO peptidases strengthened the capacity of FOG-2 to repress the GATA-4-activated BNP promoter. Lack of SUMOylation leading to increased repression activity was previously observed in the erythroid transcription factor Ikaros [23]. Conversely, additional FOG-2 SUMOylation or expression of a SUMO-1-FOG2-4KR chimeric protein abrogated the repressive function of FOG-2 and FOG-2-4KR, respectively, linking SUMO to the modulation of FOG-2-mediated transcription. How can SUMOylation restrain FOG-2′s activity? The finding that the E3 ligases examined did not increase FOG-2 SUMOylation suggested that other factors could be involved in the control of FOG-2 SUMO modification. Recent work [22] and our unpublished observation that the SUMOylation of FOG-1 is increased in the presence of GATA-1 led to the finding that FOG-2 SUMOylation is strongly enhanced by the presence of GATA-4. Moreover, the FOG-2/GATA-4 interaction is influenced by the SUMOylation state of FOG-2, with a more than 3-fold increase in the retention of GATA-4 by the mutant FOG-2-4KR molecule. This strongly suggests that the level of FOG-2 SUMOylation may be part of a regulatory loop in which GATA-4 itself modulates the activity of its co-repressor. Since SUMOylation is a dynamic and reversible modification, this could serve as a flexible mechanism to rapidly fine-tune the activity of FOG-2. This study supports the proposal that an increase in SUMOylation promotes GATA-4 transcriptional activity by up-regulating GATA-4 activation [36] and by decreasing the repression activity of FOG-2.

In summary, this study provides evidence that the biological activity of FOG-2 is dependent on the presence of intact SUMOylation sites. FOG-2 SUMO mutants served as stronger transcriptional repressors and interacted more efficiently with GATA-4. These observations suggest that SUMO modification is a crucial mechanism for FOG-2-mediated transcriptional repression. In addition, it is known that FOG-2 is essential for cardiac development [5] and that GATA-4 [36], NKX-2.5 [34], [44], p300 [21] and other cardiac proteins [45] are also targets for SUMO modification and that decreased SUMOylation can result in development of congenital heart defects [46]. All these data together with our findings place SUMOylation as a critical regulatory event of both cardiogenesis and adult cardiac function.

## Supporting Information

Figure S1 **E3 ligases or Ubc9 do not increase FOG-2 SUMOylation.** (A) COS-7 cells were transfected with a FOG-2 expression vector (left panel) or FOG-2 plus GFP-SUMO-1 (right panel) and the expression vectors indicated in the figure. Cell lysates were obtained in the presence of NEM and the proteins detected by Western blot. The presence of the SUMO E2 ligase Ubc9 in the absence (lane 2, left panel) or presence (lane 7, left panel) of co-expressed SUMO-1 did not increase FOG-2 SUMOylation. An inactive mutant of Ubc9 (Ubc9C93S) was used as negative control (lane 3, left panel). Co-expression of FOG-2 with a minimal amount of GFP-SUMO-1 plasmid (100 ng) led to weak FOG-2 SUMOylation (lane 1, right panel, arrowheads). Co-transfection of the indicated SUMO E3 ligases did not increase FOG-2 SUMOylation (lanes 2 to 5, right panel). In fact there was a decrease in FOG-2 SUMOylation in the presence of Ubc9 (lane 7, left panel) or E3 ligases (lanes 2 to 5, right panel). This is likely due to the depletion of available SUMO due to the E2- and E3-mediated increase in SUMOylation of other cellular proteins. Together, these experiments indicate that, in COS-7 cells the SUMOylation of FOG-2 is not influenced by co-expression of E2 or E3 ligases. (B) Nuclear localization in HeLa cells. HeLa cells were transfected with GFP-FOG-2 or GFP-FOG-2-4KR fusion proteins as indicated in the figure. The cell nuclei were stained with PI (red). There was no detectable difference in the sub-cellular or sub-nuclear distribution of wt and mutant FOG-2. Asterisks indicate non-specific bands detected by the FOG-2 antibody. IB, immunoblot.
(TIF)
