# Correction: SUMOylation Regulates the Transcriptional Repression Activity of FOG-2 and Its Association with GATA-4

**DOI:** 10.1371/annotation/3d7a31cd-09e7-47d1-af57-c58c7082058d

**Published:** 2013-05-17

**Authors:** José Perdomo, Xing-Mai Jiang, Daniel R. Carter, Levon M. Khachigian, Beng H. Chong

There is an error in the following sentence in the Abstract: FOG-2 SUMOylation occurs at four lysine residues (K312, 471, 915, 955). The number "K312" should be "K324". 

