# Supplementary figures and images for: SUMOylation Regulates the Transcriptional Repression Activity of FOG-2 and Its Association with GATA-4
Source: PLoS One. 2012 Nov 30;7(11):e50637. doi: 10.1371/journal.pone.0050637 (PMC3511347; doi:10.1371/journal.pone.0050637)

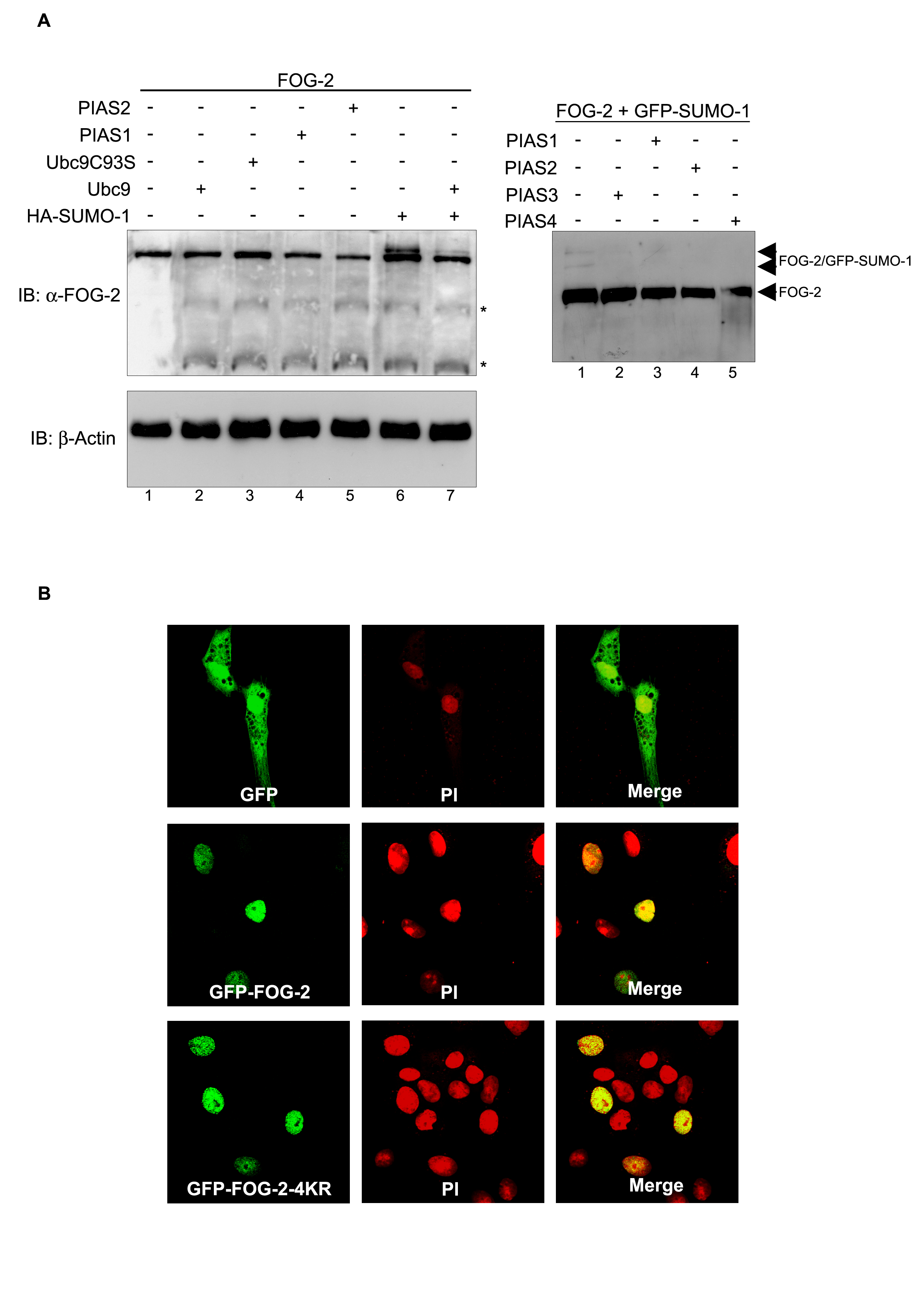

Supplement: Figure S1 — E3 ligases or Ubc9 do not increase FOG-2 SUMOylation. (A) COS-7 cells were transfected with a FOG-2 expression vector (left panel) or FOG-2 plus GFP-SUMO-1 (right panel) and the expression vectors indicated in the figure. Cell lysates were obtained in the presence of NEM and the proteins detected by Western blot. The presence of the SUMO E2 ligase Ubc9 in the absence (lane 2, left panel) or presence (lane 7, left panel) of co-expressed SUMO-1 did not increase FOG-2 SUMOylation. An inactive mutant of Ubc9 (Ubc9C93S) was used as negative control (lane 3, left panel). Co-expression of FOG-2 with a minimal amount of GFP-SUMO-1 plasmid (100 ng) led to weak FOG-2 SUMOylation (lane 1, right panel, arrowheads). Co-transfection of the indicated SUMO E3 ligases did not increase FOG-2 SUMOylation (lanes 2 to 5, right panel). In fact there was a decrease in FOG-2 SUMOylation in the presence of Ubc9 (lane 7, left panel) or E3 ligases (lanes 2 to 5, right panel). This is likely due to the depletion of available SUMO due to the E2- and E3-mediated increase in SUMOylation of other cellular proteins. Together, these experiments indicate that, in COS-7 cells the SUMOylation of FOG-2 is not influenced by co-expression of E2 or E3 ligases. (B) Nuclear localization in HeLa cells. HeLa cells were transfected with GFP-FOG-2 or GFP-FOG-2-4KR fusion proteins as indicated in the figure. The cell nuclei were stained with PI (red). There was no detectable difference in the sub-cellular or sub-nuclear distribution of wt and mutant FOG-2. Asterisks indicate non-specific bands detected by the FOG-2 antibody. IB, immunoblot. (TIF) [file pone.0050637.s001.tif]
